# Impact of Etiology on the Outcomes in Heart Failure Patients Treated with Cardiac Resynchronization Therapy: A Meta-Analysis

**DOI:** 10.1371/journal.pone.0094614

**Published:** 2014-04-14

**Authors:** Yanmei Chen, Chongyang Duan, Feng Liu, Shuxin Shen, Pingyan Chen, Jianping Bin

**Affiliations:** 1 Department of Cardiology and National Key Lab for Organ Failure Research, Nanfang Hospital, Southern Medical University, Guangzhou, China; 2 Department of Biostatistics, Southern Medical University, Guangzhou, China; Tokai University, Japan

## Abstract

**Background:**

Cardiac resynchronization therapy (CRT) has been extensively demonstrated to benefit heart failure patients, but the role of underlying heart failure etiology in the outcomes was not consistently proven. This meta-analysis aimed to determine whether efficacy and effectiveness of CRT is affected by underlying heart failure etiology.

**Methods and Results:**

Searches of MEDLINE, EMBASE and Cochrane databases were conducted to identify RCTs and observational studies that reported clinical and functional outcomes of CRT in ischemic cardiomyopathy (ICM) and non-ischemic cardiomyopathy (NICM) patients. Efficacy of CRT was assessed in 7 randomized controlled trials (RCTs) with 7072 patients and effectiveness of CRT was evaluated in 14 observational studies with 3463 patients In the pooled analysis of RCTs, we found that CRT decreased mortality or heart failure hospitalization by 29% in ICM patients (95% confidence interval [CI], 21% to 35%), and by 28% (95% CI, 18% to 37%) in NICM patients. No significant difference was observed between the 2 etiology groups (P = 0.55). In the pooled analysis of observational studies, however, we found that ICM patients had a 54% greater risk for mortality or HF hospitalization than NICM patients (relative risk: 1.54; 95% CI: 1.30–1.83; P<0.001). Both RCTs and observational studies demonstrated that NICM patients had greater echocardiographic improvements in the left ventricular ejection fraction and end-systolic volume, as compared with ICM patients (both P<0.001).

**Conclusion:**

CRT might reduce mortality or heart failure hospitalization in both ICM and NICM patients similarly. The improvement of the left ventricular function and remodeling is greater in NICM patients.

## Introduction

Cardiac resynchronization therapy (CRT) has been extensively demonstrated on improving symptoms, cardiac function and survival of heart failure (HF) patients [1]. Consequently, CRT is increasingly applied in HF treatment and its guidelines have been established [2]. However, approximately 30% of the patients who had been indicated for CRT by the guidelines still did not benefit from the treatment in clinical practice, particularly those with ischemic cardiomyopathy (ICM) [3]. Previous reports have shown that HF etiology might affect the responsiveness of CRT mainly due to variances in the pathophysiology between ICM and non-ischemic cardiomyopathy (NICM) patients [4]. Addressing whether different responses might exist between ICM and NICM patients is of great importance to the implementation of CRT in clinical practice.

A number of studies have been conducted to assess the impact of HF etiology on the response to CRT [5], [6]. However, the findings are not consistent. Some studies indicated ICM patients had similar benefits from CRT, as compared with NICM patients [7]–[9]. While in other studies, ICM patients had fewer clinical benefits [10], [11]. Because there is no consensus regarding the impact of HF etiology on CRT outcomes in HF patients, it is necessary to pool current evidence available to clarify it. Recently, a meta-analysis report based on the subgroup analysis in RCTs alone has been published by Letter Online [12]. However, it might be incomplete and underpowered to address this issue for that report to only include 5 RCTs. The present meta-analysis summarized the data available not only from large RCTs, but also from observational studies. In addition to the primary outcomes of mortality and HF hospitalization, we examined the secondary outcomes of echocardiographic and functional improvements. This meta-analysis aimed at determining whether HF etiology may affect the CRT efficacy (outcomes in RCTs) and the CRT effectiveness (outcomes in observational studies).

## Methods

This meta-analysis was performed in accordance with the preferred reporting items for systematic reviews and meta-analyses (PRISMA) and the reporting Meta-Analyses of Observational Studies in Epidemiology (MOOSE) [13], [14].

### Literature search

Systematic searches were made of multiple databases including MEDLINE, EMBASE, and Cochrane Library to retrieve all published clinical studies from 2000 through December 2012. The following search terms were used: 1) heart failure or cardiomyopathy, 2) cardiac resynchronization therapy or biventricular pacing. Our search strategy was limited to include studies involving human subjects and those published in English. Additionally, we hand searched the references cited in relevant publications.

### Study selection

To address the impact of HF etiology on CRT efficacy, we included all published RCTs of CRT that reported primary and/or secondary outcomes in subgroup analyses stratified by HF etiology. To assess the difference regarding CRT effectiveness, we included the observational studies that 1) performed a contemporaneous comparison of ICM and NICM groups focused on CRT, 2) originally reported the primary and/or secondary outcomes, 3) had more than 30 participants, and 4) had a minimum follow-up of 6 months.

When a large trial produced multiple publications, we retrieved the most complete dataset; when the data was not detailed enough, we contacted the authors to get the necessary information.

### Outcomes definitions

The primary outcomes were mortality or HF hospitalization. The secondary outcomes were the left ventricular function and size measured by echocardiography (i.e., left ventricular ejection fraction [LVEF] and left ventricular end-systolic volume [LVESV]), exercise capacity (6-min walk test [6-MWD]) and quality of life (QOL) measured by the Minnesota Living with Heart Failure questionnaire in which lower scores indicate an improvement.

### Data extraction

Two reviewers (Chen and Liu) independently extracted data by using standard data extraction forms. The extracted information included study and patient characteristics, primary and secondary outcomes, and study quality. Discrepancies were resolved by a consensus.

### Methodological quality

The quality of RCTs included was assessed by Jadad quality scale [15], and the quality of observational studies was assessed by Newcastle-Ottawa Scale tool (available at: http://www.ohri.ca/programs/clinical_epidemiology/oxford.asp).

### Statistical analysis

For the primary outcomes, relative risks (RRs) and 95% confidence interval (CI) were used as the measure across studies. Hazard ratios (HRs) were reported to be broadly equivalent to RRs [16]–[18]. If the included studies provided effect estimates such as HRs, they were directly used in the pooled analysis. If the effect estimates were not available in the studies, the RRs were calculated by using the following formula: RR =  Probability of events in the ICM group/Probability of events in the NICM group. We performed sensitivity analyses to examine the robustness of the results regarding primary outcomes. For analysis of the secondary outcomes, we calculated the weighted mean difference [WMD] (i.e., LVEF, 6-MWD and QOL) or standard mean difference [SMD] (LVESVE) to the pooled analysis. All results were reported with 95% CI.

Statistical heterogeneity was tested by the Cochran Q statistic and reported as I^2^. Fixed-effects model was used. If a substantial heterogeneity (I^2^>50%) was found, then a random-effect model was used. The possibility of publication bias was assessed by Begg's test. A two-sided *p*-value<0.05 was considered statistically significant. Data was analyzed using the statistical program Stata (version 11.2, Stata Corp, College Station, Texas) and R software (version. 3.0.1, available at: http://www.r-project.org/).

## Results

### Search results

**Figure 1:**
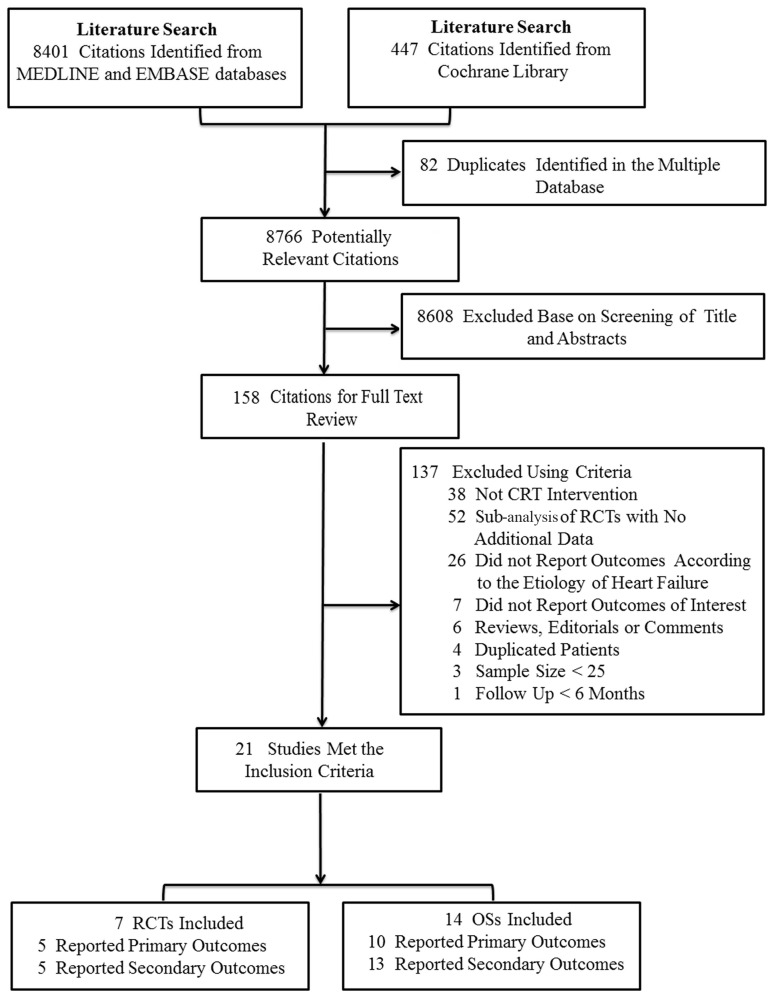
Flowchart of Studies Selection for Meta-analysis. CRT, cardiac resynchronization therapy; OSs, observational studies; RCT, randomized controlled trial.

The flow diagram of literature search and study selection is displayed in Figure 1. Our initial search yielded 8401 citations from MEDLINE and EMBASE databases, and 447 citations from Cochrane Library. Of those, 21 studies met the selection criteria, including 7 RCTs[7], [8], [19]–[23] and their sub-analysis [24]–[28] for assessing CRT efficacy, and 14 observational studies[10], [11], [29]–[39] for assessing CRT effectiveness.

### Study characteristics of RCTs

The study characteristics of included RCTs are displayed in Table 1. A total of 7072 HF patients were randomly assigned to CRT group (or CRT plus implantable cardioverter-defibrillator (ICD), n = 4280) and control group (ICD only or medical therapy (MT) only, n = 2792). The COMPANION [7] was a three-armed randomized, controlled trial, in which the patients were randomly assigned to medical therapy group, CRT group, or CRT-D group. Both the subgroup with CRT-D verse medical therapy and the subgroup with CRT verse medical therapy were included in our meta-analysis. Mean length of follow-up ranged from 11.9 to 40 months. The primary outcomes reported in subgroup analyses according to HF etiology always included mortality or HF hospitalization despite a few variations across these trials. All RCTs were multicenter, randomized controlled trials. The MIRACLE [20], [25], REVERSE [21], [27], and RAFT [23] trials were double-blinded, whereas the other four were single-blinded. All trials had a blinded outcomes assessor. In summary, the quality of all the RCTs included was high (Jadad scores≥4 in all RCTs). The patients assigned to CRT (or CRT-D) group verse control group were well balanced in age, sex, ejection fraction, QRS duration, and medication use. The baseline characteristics of patients enrolled in the RCTs are listed in Table S1.

**Table 1:** Characteristics and Quality of Randomized Controlled Trials included in the Meta-analysis.

Study (Year)	N	Inclusion criteria			Design		Mean follow-Up (m)	Primary endpoints	Secondary endpoints	Jadad score
		NYHA class	LVEF(%)	QRS (ms)	Intervention(n)	Control (n)				
**MUSTIC (2001)^19,24^**	58	III	≤35	≥150	CRT(n = 29)	MT (n = 29)	12	NA	LVESV, 6-MWD	4
**MIRACLE (2002) ^20,25^**	453	III or IV	≤40	≥130	CRT(n = 228)	MT (n = 225)	12	NA	LEVF, LVESV, 6-MWD,QOL	5
**COMPANION (2004)^6^** *	1520	III or IV	≤35	≥120	CRT-D (n = 595) or CRT(n = 617)	MT (n = 308)	11.9 to 16.2	i)Death ii)hospitalization for any cause	NA	4
**CARE-HF (2005)^7,26^**	813	III or IV	≤35	≥120	CRT (n = 409)	MT (n = 404)	29.4	i)Deathii)unplanned hospitalization for HF	LVEF, LVESV	4
**REVERSE (2008)^21,27^**	610	I or II	≤40	≥120	CRT-on (n = 419)	CRT-off (n = 191)	12	i)Death ii)hospitalization iii)worsened HF	LVEF, LVESV, 6-MWD,QOL	5
**MADIT-CRT (2009)^22,28^**	1820	I or II	≤30	≥130	CRT-D (n = 1089)	ICD (n = 731)	28.8	i)Death ii)nonfatal HF	LVEF, LVESV	4
**RAFT (2010)^23^**	1798	II or III	≤30	≥120	CRT-D (n = 894)	ICD (n = 904)	40	i)Death ii)hospitalization for HF	NA	5

*COMPANION trial was 3 arms (CRT-D versus CRT versus medical therapy) design. 6-MWD, 6-min walking distance; ICD, implantable cardioverter-defibrillator; LVEF, left ventricular ejection fraction; LVESV, left ventricular end systolic volume; MT, medical therapy; NA, not available; NYHA, New York Heart Association; QOL, Quality of Life.

### Study characteristics of observational studies

The pooled study characteristics of observational studies are displayed in Table 2. Of the 14 observational studies included, 11 studies had prospective cohort design and 3 studies had retrospective cohort design. Among a total of 3463 HF patients, 1842 were in ICM group and 1621 in NICM group. The mean follow-up period, ranging from 6 to 52.8 months, was similar in the ICM and NICM groups. Quality of the included observational studies was assessed by Newcastle-Ottawa Scale tool was high (median score, 7; range, 6 to 8; Table S2). The baseline characteristics of patients enrolled in the observational studies are listed in Table S3.

**Table 2:** Characteristics of Observational Studies Included in the Meta-analysis.

Study(Year)	Study Type	Inclusion Criteria			Sample Size (N)		Mean follow-up (m)	Primary Outcomes	Secondary Outcomes
		NYHA class	QRS (ms)	LVEF (%)	ICM	NICM			
**Gasparini M (2003)^9^**	prospective	II to IV	>110	<40	75	83	11.2	Death	LVEF, LVESV, 6-MWD,QOL
**Molhoek SG(2004)^29^**	prospective	III or IV	>120	<35	34	40	ICM14.2 NICM13.8	Death from any cause	LVEF,6-MWD,QOL
**Leclercq C (2004)^30^**	prospective	III or IV	>150	<35	48	55	12	Death from any cause	6-MWD,QOL
**Waggoner, A.D (2006)^10^**	prospective	III or IV	>150	<35	19	38	20	i)Cardiac-related death or heart transplant, ii)Hospitalization due to HF	LVEF, LVESV, 6-MWD
**Soliman, O. I (2007)^31^**	prospective	III or IV	>120	<35	36	38	24	i)Cardiac-related death, ii)hospitalization due to HF	NA
**D'Andrea, A(2007)^32^**	prospective	III or IV	>120	<35	43	47	6	NA	LVEF
**Vidal, B (2007)^33^**	prospective	III or IV	>120	≤35	43	63	12	Death or heart transplant	LVEF, LVESV
**Di Biase L (2008)^11^**	prospective	III or IV	>120	≤35	219	179	52.8	Combined for death and heart transplant	LVEF
**Marsan, N. A(2009)^34^**	prospective	III or IV	>120	≤35	135	87	6	NA	LVEF, LVESV
**Boriani,G (2009)^35^**	prospective	II to IV	>130	≤35	737	635	16	Death from any cause or urgent heart transplantation	LVEF, LVESV
**Zhang, Q (2009)^36^**	prospective	III or IV	>120	<40	52	67	39	i)Death, ii)Cardiovascular hospitalization	LVEF,LVESV, 6-MWD,QOL
**Kazemi S.A(2009)^37^**	retrospective	III or IV	>125	<35	48	35	6	NA	LVEF
**Mcleod CJ (2011)^38^**	retrospective	III or IV	>120	≤35	312	191	7.1	Death	LVEF, LVESV
**Zaca, V (2011)^39^**	retrospective	III or IV	>120	≤35	41	63	12	NA	LVEF, LVESV

ICM, ischemic cardiomyopathy; NICM, non-ischemic cardiomyopathy; other abbreviations as in Table 1.

### Primary outcomes

**Figure 2:**
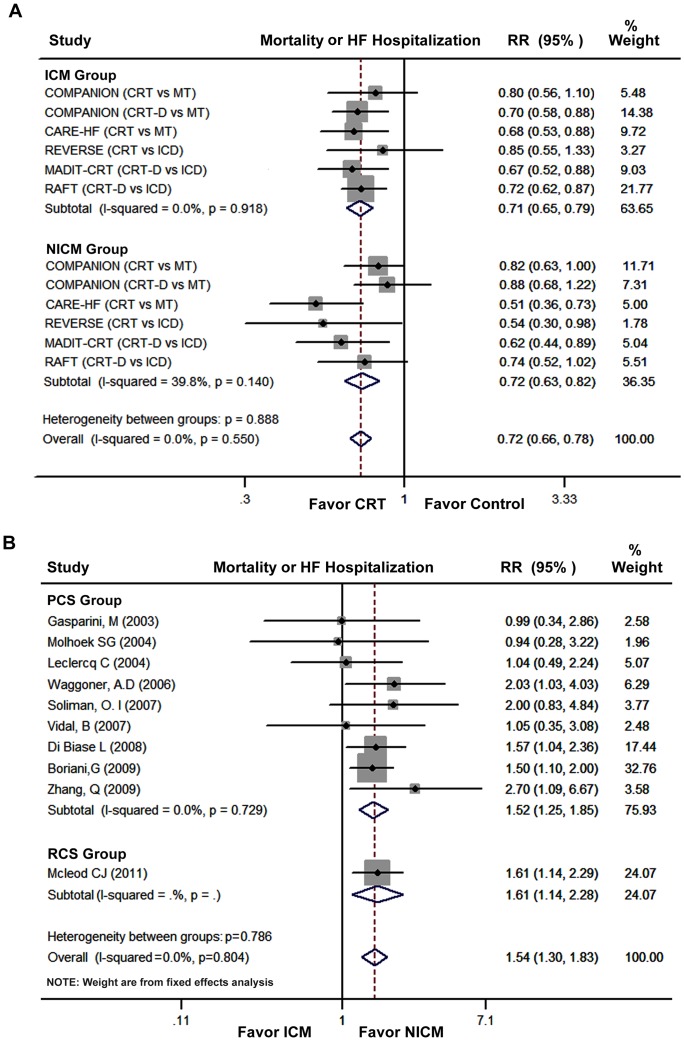
Forest Plots Showing the Impact of HF Etiology on Mortality or HF Hospitalization. (A): data from RCTs; (B): data from observational studies. COMPANION trial was 3 arms design; CI, confidence interval; HF, heart failure; ICD, implantable cardioverter-defibrillator; ICM, ischemic cardiomyopathy; MT, medical therapy; NICM, non-ischemic cardiomyopathy; PCS, prospective cohort studies; RCS, retrospective cohort studies; RR, relative risk; and other abbreviations as in Figure 1.

The impact of HF etiology on CRT efficacy in terms of the primary outcomes in RCTs is shown in Figure 2A. There are 5 RCTs that reported the primary outcomes. In 4 trials, the ICM patients who received CRT had a significant reduction in risk for mortality or HF hospitalization. Similarly, the NICM patients exhibited a significant reduction in risk for mortality or HF hospitalization in 3 trials. ICM patients randomized to the CRT (or CRT-D) group had a 29% reduction in risk for mortality or HF hospitalization (95% CI, 0.65 to 0.79; P<0.001), while a 28% reduction in the primary outcomes was noted in NICM patients (95% CI, 0.63 to 0.82, P<0.001). When the direct comparison of CRT efficacy performed using the heterogeneity test, there was no statistical difference between ICM and NICM patients (P = 0.55). No evidence for publication bias was found in both ICM and NICM group by Begg's test (P = 0.71, P = 0.06, respectively), and by visual inspection of funnel plots that were almost symmetric (Figure S1).

The impact of HF etiology on primary outcomes in observational studies is presented in Figure 2B. Among 10 observational studies that reported the primary outcomes, five studies found that ICM patients had a greater risk for mortality or HF hospitalization than NICM patients. The pooled analysis of prospective cohort studies (9 studies) showed that ICM patients had a 52% greater risk for mortality or HF hospitalization than NICM patients (95% CI, 1.25 to 1.85; P<0.001). Overall, there was a 54% greater risk for mortality or HF hospitalization in ICM patients versus NICM patients (95% CI, 1.30 to 1.83; P<0.001). In addition, a greater benefit in NICM patients remained regarding all-cause mortality (RR, 1.46; 95% CI, 1.22 to 1.71; P<0.001). No evidence of publication bias was observed in the observational studies by Begg's test (P = 0.72), and funnel plots were almost symmetric (Figure S2).

### Secondary outcomes

**Figure 3:**
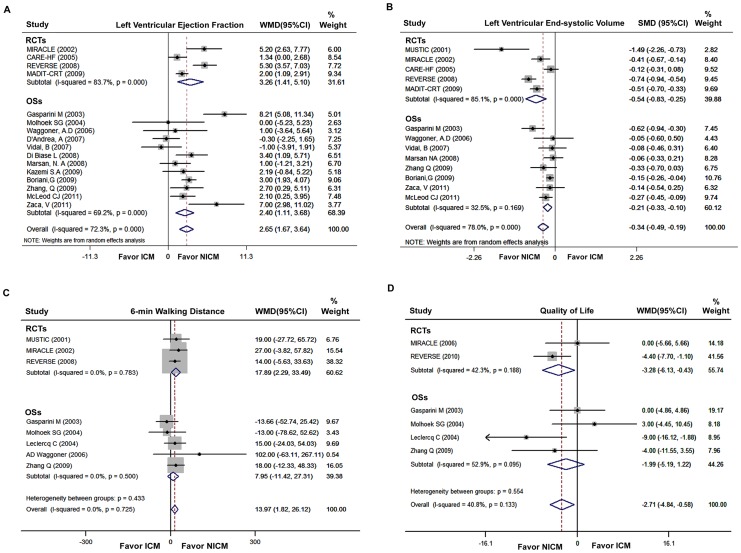
Pooled Analyses of Secondary Outcomes (the Change from Baseline) in NICM Group versus ICM Group. WMD in change of Left Ventricular Ejection Fraction (A), SMD in change of Left Ventricular End-systolic Volume (B), WMD in change of 6-Min Walking Distance (C), and WMD in change of Quality of Life (D). WMD, weighted mean difference; SMD, standardized mean difference; and other abbreviations as in Figure 2.

The pooled analysis results of LVEF and LVESV are displayed in Figure 3. Of 21 studies included, 4 RCTs and 12 observational studies provided the data of LVEF. On meta-analysis, NICM patients assigned to CRT (or CRT-D) had greater increase in LVEF (WMD: 2.65% greater, 95% CI: 1.67% to 3.64%, p<0.001, Figure 3A), as compared with ICM patients. Similarly, a reduction in LVESV was significantly greater for NICM patients than ICM patients (SMD: −0.34, 95% CI: −0.49 to −0.19, p<0.001, Figure 3B). SMD is a ratio that expresses the size of intervention effect in each study relative to the variability observed in that study.

The pooled analyses of 6-MWD and QOL are displayed in Figure 3 C and D, respectively. Compared with ICM patients, the NICM patients who received CRT had a significantly greater increase in 6-MWD (WMD, 17.89 m; 95% CI, 2.29 to 33.49 m; P<0.05), and a significant decrease in QOL scores (WMD, −3.28 score; 95% CI, −6.13 to −0.43 score; P<0.05; Lower scores of QOL indicate an improvement) by the pooled analysis of RCTs. While, these results were not confirmed in the meta-analysis of observational studies that provided the data (6-MWD: P = 0.42; QOL: P = 0.22).

### Sensitivity analyses

**Figure 4:**
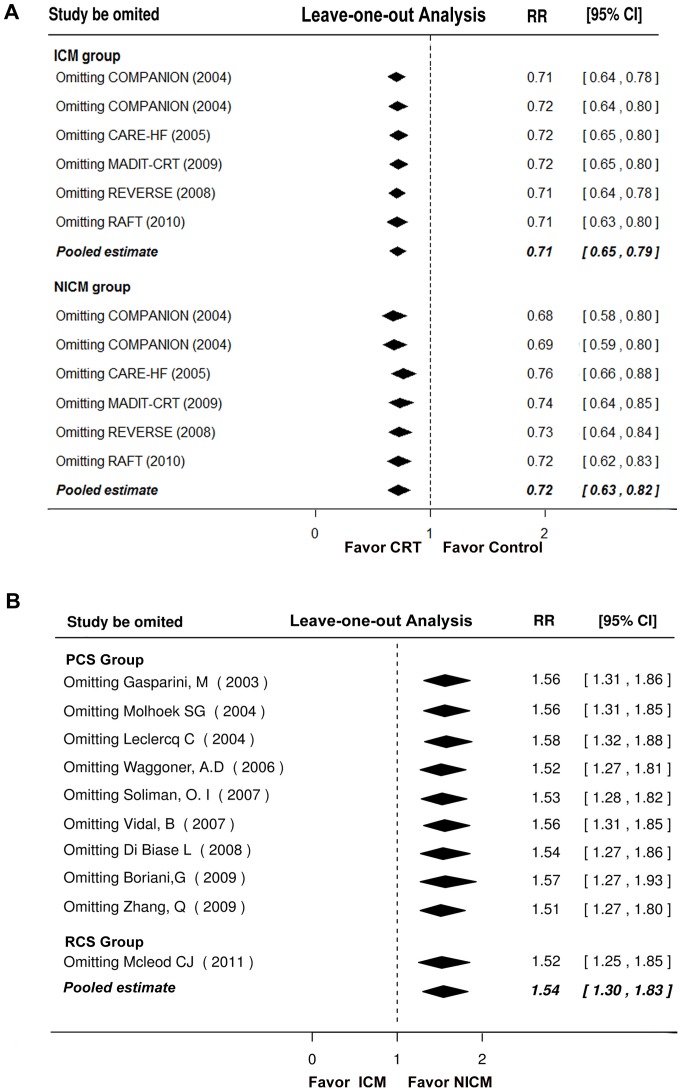
Leave-One-Out Analyses of Primary Outcomes. (A): data from RCTs; (B): data from observational studies. The RR and CIs for each row was presented as the overall effect size if that study were excluded. Abbreviations as in Figure 2.

We performed leave-one-out sensitivity analyses in both RCTs and observational studies, and found that none of the individual study significantly influenced the pooled estimate for primary outcomes (Figure 4). In addition, when the analysis was limited to those studies uniformly provided HRs, there remained a similar result (Figure S3 and S4). When the pooled analysis was limited to studies without ICD therapy (CRT versus medical therapy), statistically significant benefits of CRT were found in both ICM and NICM patients (Figure S5A). When the analysis was limited to the studies of ICD therapy in both arms (CRT-D versus ICD), the benefits of CRT-D were observed again in ICM and NICM patients (Figure S5B).

## Discussion

In this meta-analysis focused on the role of HF etiology in CRT efficacy and effectiveness, the data of 7 RCTs demonstrated a definite efficacy of CRT therapy in reduction of mortality or HF hospitalization in both ICM and NICM patients without significant difference between the 2 etiology groups. The evidence of 14 observational studies, however, showed that NICM patients exhibited a lower risk for mortality or HF hospitalization as compared with ICM patients. Interestingly, both the evidence from RCTs and observational studies revealed that improvements of LVEF and LVESV favored NICM patients.

Consistent with previous meta-analysis findings based on the subgroup analysis in RCTs [12], our meta-analysis of RCTs also revealed a similar CRT efficacy regarding mortality or HF hospitalization in ICM and NICM patients. It should be pointed out that only the subgroup data were available to the pooled analysis in this study since the current RCTs were not specially designed to determine the role of HF etiology on the outcomes of CRT. However, the credibility of subgroup analyses could be improved if the trial were large enough and the difference of a treatment effect in subgroup were assessed by the statistic tests of interaction [40]. The included 5 multicenter RCTs (in which the data of the primary outcomes were available) with 6561 patients were large enough and used the statistic tests of interaction, which provided the convincing evidence. Furthermore, the RCTs with a randomized control group could distinguish the treatment effect of CRT from the impact of natural history of ischemic diseases. Theoretically ICM patients might gain less benefit from CRT compared with the NICM patients, for ICM patients with scar burden had fewer viable cardiomyocytes to substrate for response to CRT [4]. The data of RCTs in this meta-analysis suggested that benefits of CRT in ICM patients were similar to those in NICM patients. Therefore, the absolute reduction of mortality or HF hospitalization derived from CRT was likely to be greater in ICM patients.

The results of RCTs and observational studies regarding mortality or HF hospitalization were not completely consistent with each other. Interpretation of these results required some caution. We should not overemphasize the result of observational studies for drawing the conclusion that NICM exhibited greater benefit, for the observational studies could not gauge whether the ischemic etiology really affected the benefits of CRT. Without a control group (no CRT therapy), the observational studies could not distinguish the real differences between the effect of the ischemic etiology and the effect of the intervention, although the observational studies included in this meta-analysis were specially designed for the direct comparisons between ICM and NICM groups. In addition, previous studies have demonstrated that a poorer outcome was noted in ICM patients as compared with NICM patients, which was associated with the effect of the disease itself but not with the effect of the medical therapy [41], [42]. It has been reported that the poor outcomes of ICM patients were directly related to the large scar burden and fewer viable cardiomyocytes [43]. Thus, fewer benefits observed in ICM patients were probably associated with the disease itself rather than reduced efficacy of CRT therapy.

The data of both RCTs and observational studies in secondary outcomes of CRT demonstrated that ICM patients experienced less improvement of LV function and remodeling compared with NICM patients. These findings are not surprising because ICM patients might have fewer viable cardiomyocytes to substrate for improvement in LV function after CRT [5]. In addition, too much scar tissue of ICM patients in the region of the LV pacing lead might result in no response to CRT due to inadequate pacing [5]. However, the less improvement gained by ICM patients may not be necessarily associated with lower reduction in mortality or HF hospitalization, especially in all-cause mortality. It is likely that the protective effect of CRT on mortality for ICM patients might derive mainly from the reduction of arrhythmic mortality and not merely from LV remodeling [26].

### Study limitations

This meta-analysis has several limitations. First, our selection criteria were restricted to published studies in English and might produce language bias. However, the extent of this bias should be similar in the 2 groups, allowing for the comparison of benefit of CRT. Second, there were 2 types of effect estimates (HRs and RRs) for primary outcomes in studies included. Given the similarities of these 2 effect estimates, the HRs were directly used in the pooled analysis. We addressed this limitation by performing sensitivity analysis, which yielded similar results. Third, the primary outcome reported in subgroup analyses according to HF etiology varied across the included studies. However, all-cause mortality and heart failure hospitalization were always included. The pooled results also showed that the significant reduction in any of the composite outcomes was observed in both ICM and NICM patients after CRT.

### Conclusion

In summary, this meta-analysis showed that CRT significantly reduced mortality and HF hospitalizations and improved LVEF and LVESV in both ICM and NICM patients. The effect of CRT on mortality or HF hospitalization may be similar in ICM and NICM patients, despite less improvement in LV function and remodeling was noted in ICM patients. Our findings suggest that both ICM and NICM patients should not deny the intervention of CRT.

## Supporting Information

Figure S1 **Begg's Funnel Plots with Pseudo 95% Confidence Limits in RCTs.** (A): ICM group; (B): NICM group. ICM indicates ischemic cardiomyopathy; NICM, non-ischemic cardiomyopathy; RCTs, randomized controlled trials, RR, relative risk; and SE standard error.
(TIF)

Figure S2 **Begg's Funnel Plot with Pseudo 95% Confidence Limits in Observational studies.** OSs indicates observational studies; RR, relative risk; and SE standard error.
(TIF)

Figure S3 **Sensitivity Analysis of RCTs.** (A): ICM group; (B): NICM group. The HR group was the studies that directly provided HRs for pooling analysis, and the RR group was those studies calculated by using primary data for pooling analysis. CI, indicates confidence interval; CRT, cardiac resynchronization therapy; HF, heart failure; HR, Hazard ratios; ICM, ischemic cardiomyopathy; NICM, non-ischemic cardiomyopathy; RCTs, randomized controlled trials, and RR, relative risk.
(TIF)

Figure S4 **Sensitivity Analysis of Observational studies.** The HR group was the studies that directly provided HRs for pooling analysis, and the RR group was those studies calculated by using primary data for pooling analysis. CI, indicates confidence interval; HF, heart failure; HR, Hazard ratios; ICM, ischemic cardiomyopathy; NICM, non-ischemic cardiomyopathy; OSs, observational studies; and RR, relative risk.
(TIF)

Figure S5 **Sensitivity Analysis of RCTs.** (A): CRT group verse Medical therapy group; (B): CRT group verse ICD group. CI, indicates confidence interval; CRT, cardiac resynchronization therapy; HF, heart failure; HR, Hazard ratios; ICD, implantable cardioverter-defibrillator; ICM, ischemic cardiomyopathy; NICM, non-ischemic cardiomyopathy; RCTs, randomized controlled trials, and RR, relative risk.
(TIF)

Table S1 **Characteristics of Patients enrolled in Randomized Controlled Trials.**
(DOCX)

Table S2 **The Quality of Observational Studies Assessed by Newcastle–Ottawa Scale.**
(DOCX)

Table S3 **Characteristics of patients enrolled in Observational Studies.**
(DOCX)

Checklist S1 **PRISMA Checklist.**
(DOCX)
