# Supplementary figures and images for: Impact of Etiology on the Outcomes in Heart Failure Patients Treated with Cardiac Resynchronization Therapy: A Meta-Analysis
Source: PLoS One. 2014 Apr 14;9(4):e94614. doi: 10.1371/journal.pone.0094614 (PMC3986107; doi:10.1371/journal.pone.0094614)

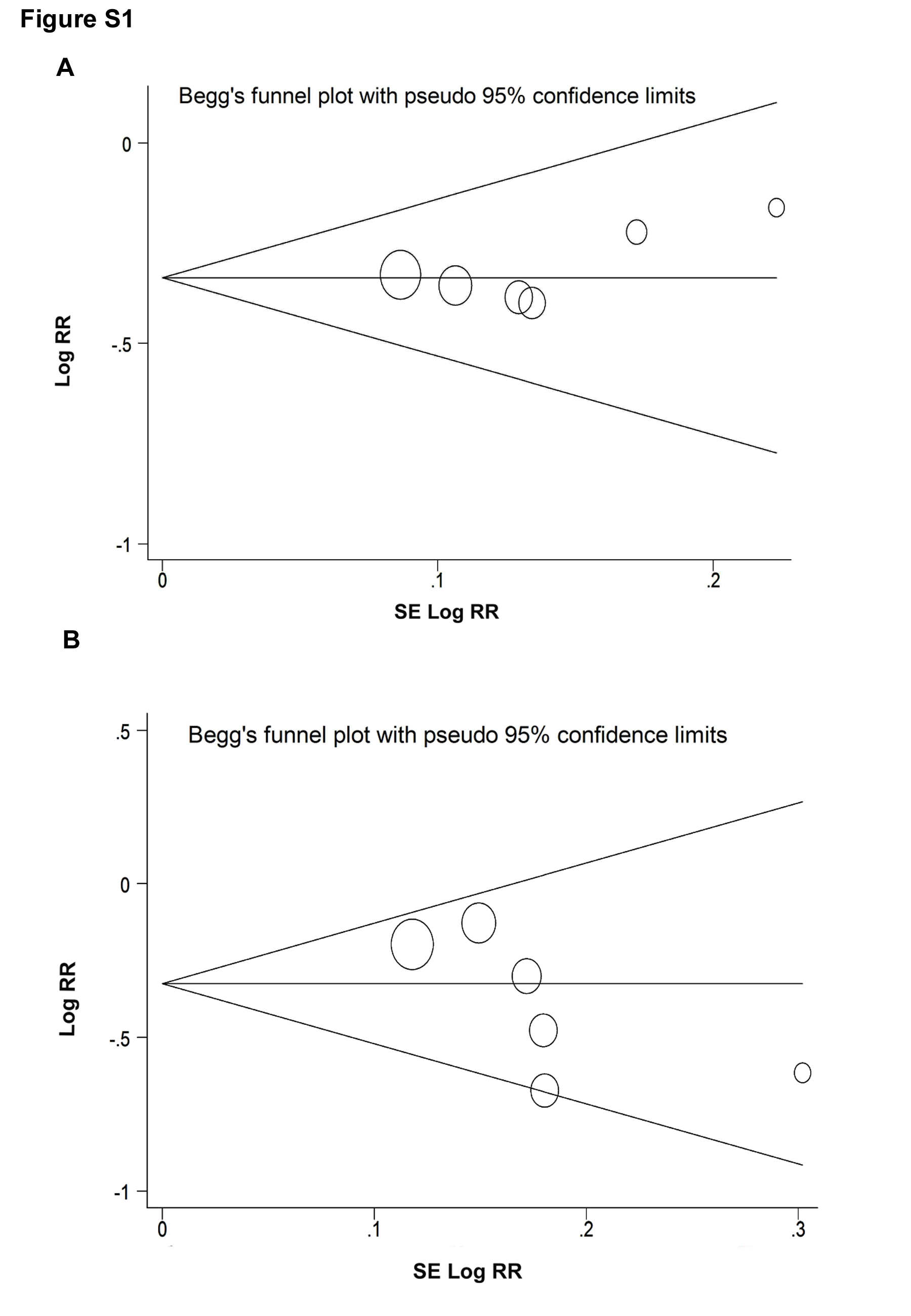

Supplement: Figure S1 — Begg's Funnel Plots with Pseudo 95% Confidence Limits in RCTs. (A): ICM group; (B): NICM group. ICM indicates ischemic cardiomyopathy; NICM, non-ischemic cardiomyopathy; RCTs, randomized controlled trials, RR, relative risk; and SE standard error. (TIF) [file pone.0094614.s001.tif]

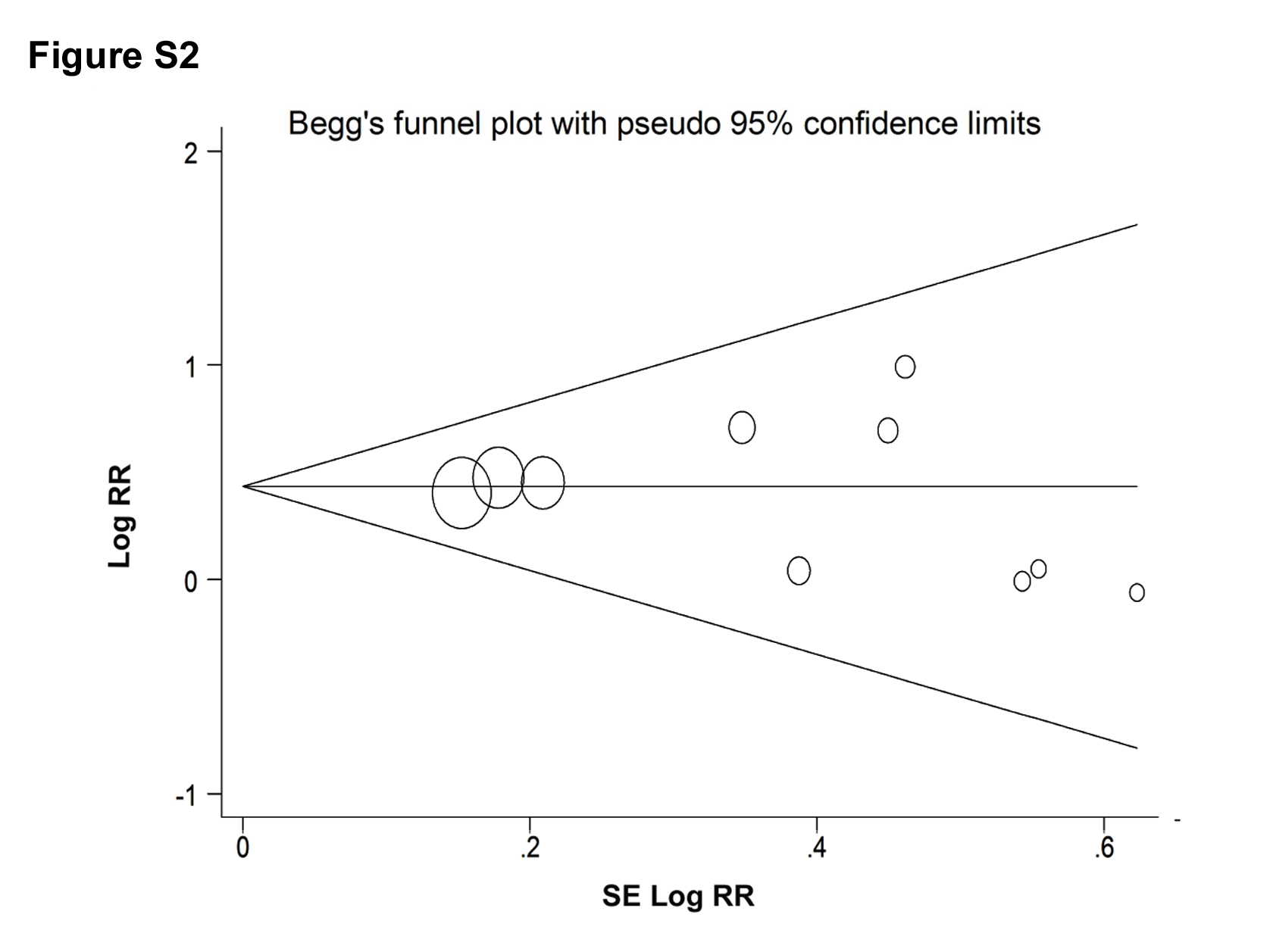

Supplement: Figure S2 — Begg's Funnel Plot with Pseudo 95% Confidence Limits in Observational studies. OSs indicates observational studies; RR, relative risk; and SE standard error. (TIF) [file pone.0094614.s002.tif]

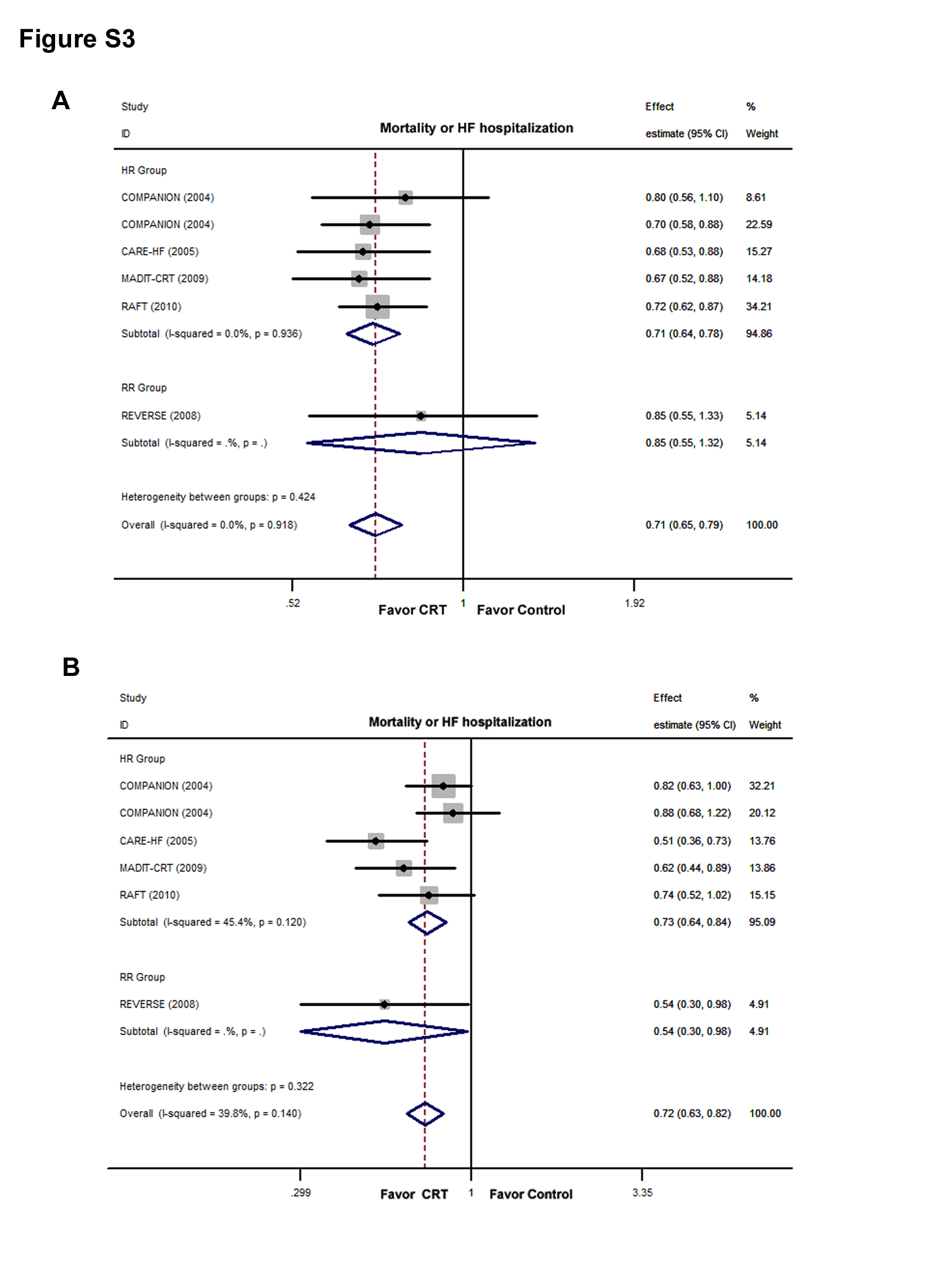

Supplement: Figure S3 — Sensitivity Analysis of RCTs. (A): ICM group; (B): NICM group. The HR group was the studies that directly provided HRs for pooling analysis, and the RR group was those studies calculated by using primary data for pooling analysis. CI, indicates confidence interval; CRT, cardiac resynchronization therapy; HF, heart failure; HR, Hazard ratios; ICM, ischemic cardiomyopathy; NICM, non-ischemic cardiomyopathy; RCTs, randomized controlled trials, and RR, relative risk. (TIF) [file pone.0094614.s003.tif]

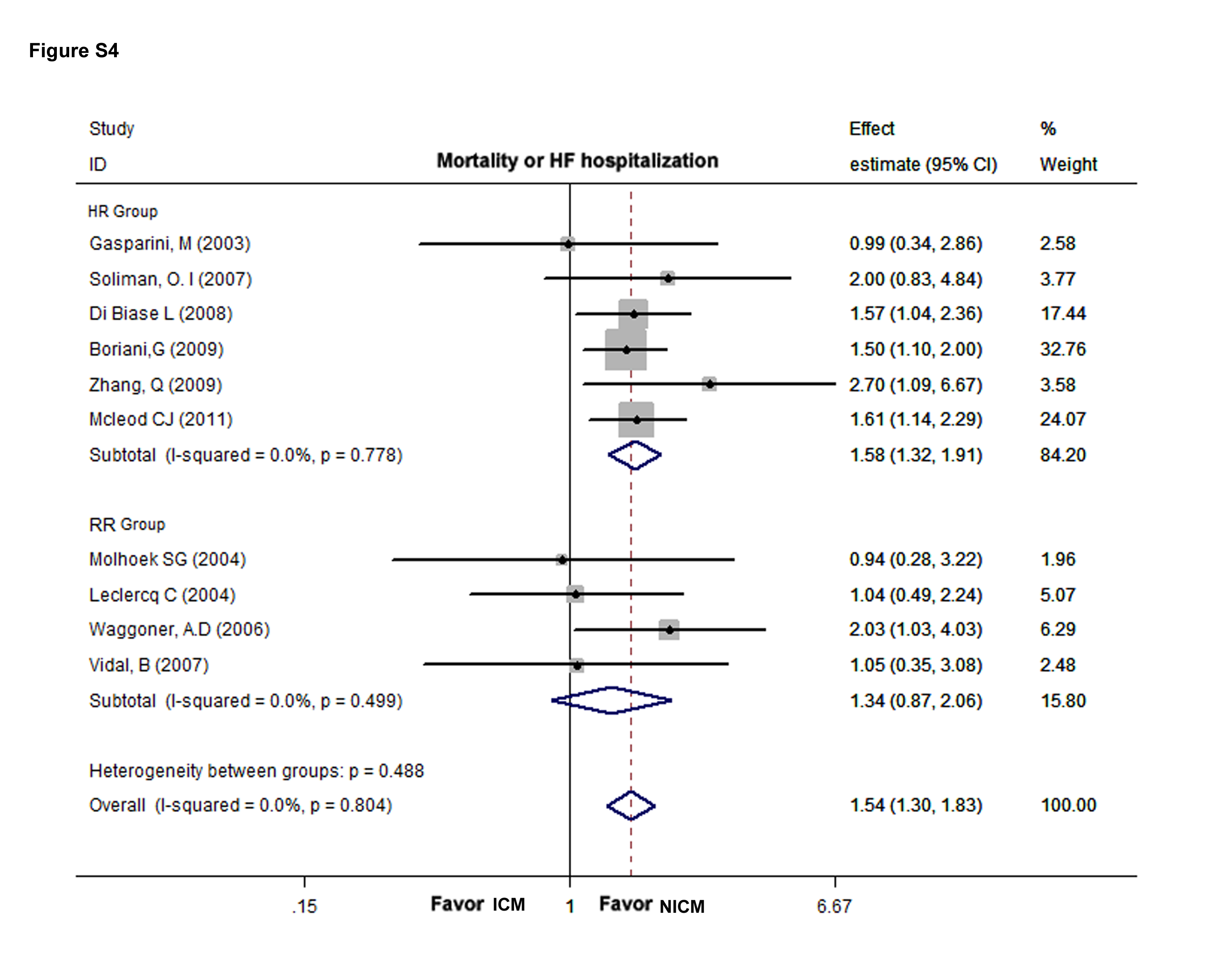

Supplement: Figure S4 — Sensitivity Analysis of Observational studies. The HR group was the studies that directly provided HRs for pooling analysis, and the RR group was those studies calculated by using primary data for pooling analysis. CI, indicates confidence interval; HF, heart failure; HR, Hazard ratios; ICM, ischemic cardiomyopathy; NICM, non-ischemic cardiomyopathy; OSs, observational studies; and RR, relative risk. (TIF) [file pone.0094614.s004.tif]

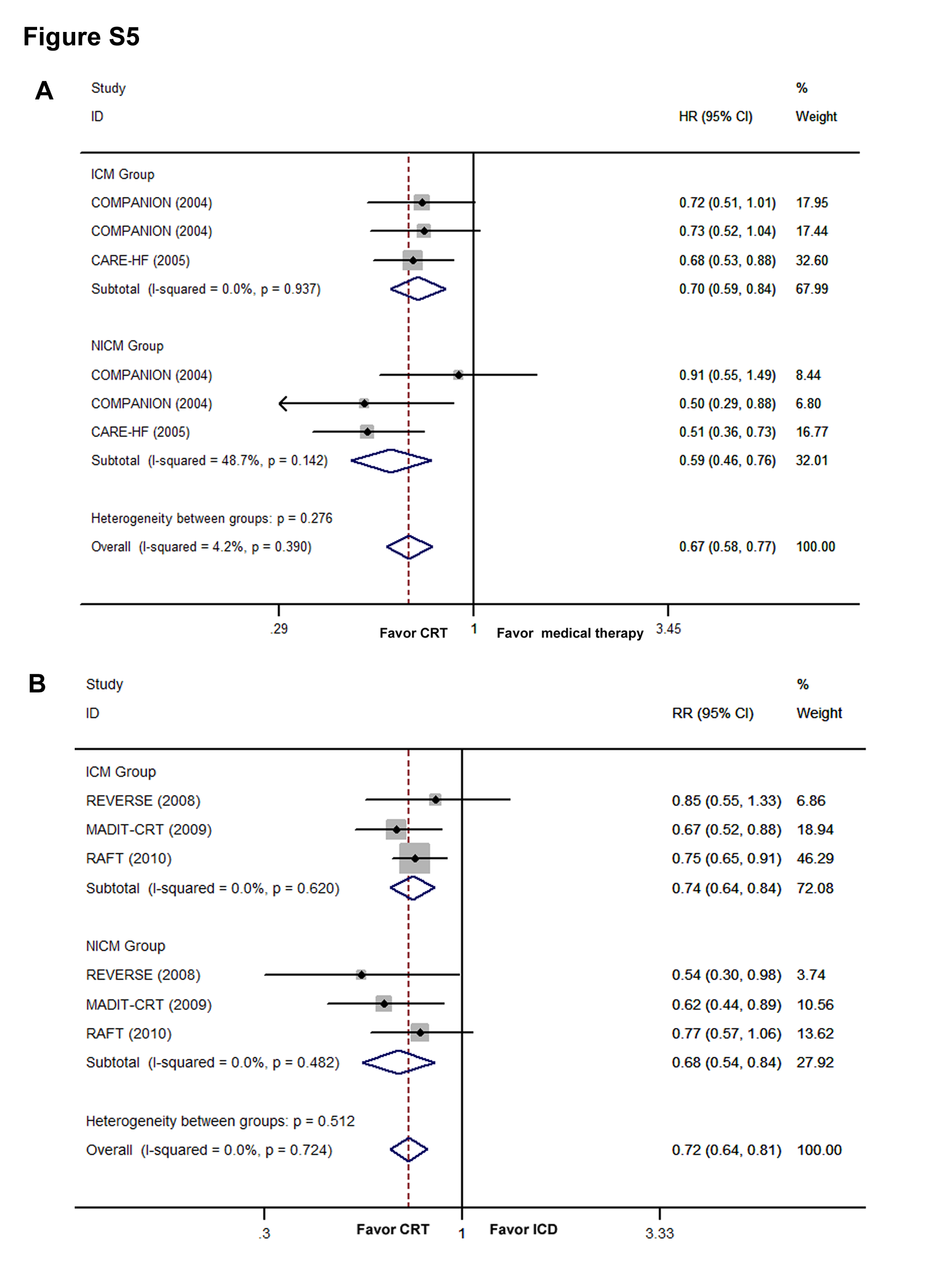

Supplement: Figure S5 — Sensitivity Analysis of RCTs. (A): CRT group verse Medical therapy group; (B): CRT group verse ICD group. CI, indicates confidence interval; CRT, cardiac resynchronization therapy; HF, heart failure; HR, Hazard ratios; ICD, implantable cardioverter-defibrillator; ICM, ischemic cardiomyopathy; NICM, non-ischemic cardiomyopathy; RCTs, randomized controlled trials, and RR, relative risk. (TIF) [file pone.0094614.s005.tif]
