# Supplementary material for: Impact of Etiology on the Outcomes in Heart Failure Patients Treated with Cardiac Resynchronization Therapy: A Meta-Analysis
Source: PLoS One. 2014 Apr 14;9(4):e94614. doi: 10.1371/journal.pone.0094614 (PMC3986107; doi:10.1371/journal.pone.0094614)
Supplement: Table S1 — Characteristics of Patients enrolled in Randomized Controlled Trials. (DOCX) [file pone.0094614.s006.docx]

| **Study (Year)** | **MUSTIC(2001)^19,24^** | **MIRACLE (2002)^20,25^** | **COMPANION (2004) ^6^** | | **CARE-HF (2005)^7,26^** | **REVERSE (2008) ^21,27^** | **MADIT-CRT (2009) ^22,28^** | **RAFT (2010) ^23^** |
| --- | --- | --- | --- | --- | --- | --- | --- | --- |
|  |  |  |  |  |  |  |  |  |
| **Definition of ICM** | i)Significant CAD (>70% stenosis) by CA,ii) Prior MI. | i) Prior MI,ii) Prior PCI or CABG | NA | | i)CA indicating major diseases, ii) Prior MI, iii) Prior CABG or PCI. | i) Evidence of 2- or 3-vessel disease by CA, ii) Prior MI, iii) Coronary revascularization. | i) Signiﬁcant CAD at CA, ii) Angina pectoris or other sym; ptoms or signsiii) Prior MI, iv) Prior coronary revascularization | NA |
|  |  |  |  |  |  |  |  |  |
|  |  |  |  |  |  |  |  |  |
|  |  |  |  |  |  |  |  |  |
| **Subgroups** |  |  |  | |  |  |  |  |
| **ICM(n)** | 16 | 115 | 837 | | 443 | 270 | 999 | 1201 |
| **NICM(n)** | 18 | 113 | 682 | | 370 | 217 | 821 | 597 |
|  |  |  | CRT | CRT-D |  |  |  |  |
| **Mean age (y)** | 63 | 63.9 | 67 | 66 | 67 | 63 | 65 | 66 |
| **Males (%)** | 75 | 68.4 | 67 | 67 | 73 | 75 | 75 | 83 |
| **Diabetes(%)** | NA | NA | 39 | 41 | 41 | 30 | 23 | 33 |
| **Atrial ﬁbrillation (%)** | 0 | 0 | 0 | 0 | 0 | 0 | 12 | 13 |
| **LBBB (%)** | NA | NA | 69 | 73 | 90 | NA | 70 | 72 |
| **RBBB (%)** | NA | NA | 12 | 10 | 5 | NA | 13 | 9 |
| **Drug** |  |  |  |  |  |  |  |  |
| **Beta-blockers (%)** | NA | 62 | 68 | 68 | 72 | 95 | 93 | 90 |
|  |  |  |  |  |  |  |  |  |
| **ACEI/ARBS (%)** | NA | 93 | 89 | 90 | 95 | 97 | 98 | 97 |
|  |  |  |  |  |  |  |  |  |
| **Digoxin (%)** | NA | NA | NA | NA | 56 | NA | 26 | 36 |
| **Spironolactone (%)** | NA | NA | 53 | 55 | 56 | NA | 32 | 42 |
|  |  |  |  |  |  |  |  |  |
| **Diuretic(%)** | NA | 94 | 94 | 97 | 43 | 80 | 75 | 84 |

**Table S1: Characteristics of Patients enrolled in Randomized Controlled Trials**

6-MWD indicates 6-min walking distance; ACE-I, angiotensin-converting enzyme inhibitor; ARBs, angiotensin-receptor blockers; CA, coronary angiography; CABG, coronary artery bypass grafting; CAD, coronary artery diseases; CI, confidence interval; ICD, implantable cardioverter-defibrillator; ICM, ischemic cardiomyopathy; LBBB, left bundle branch block; LVEF, left ventricular ejection fraction; LVESV, left ventricular end systolic volume; MT, medical therapy; MI, myocardial infarction; NA, not available; NICM, non-ischemic cardiomyopathy; NYHA, New York Heart Association; PCI, percutaneous coronary intervention; QOL, Quality of Life; and RR, relative risk; and RBBB, right bundle branch block.
