# Supplementary material for: Impact of Etiology on the Outcomes in Heart Failure Patients Treated with Cardiac Resynchronization Therapy: A Meta-Analysis
Source: PLoS One. 2014 Apr 14;9(4):e94614. doi: 10.1371/journal.pone.0094614 (PMC3986107; doi:10.1371/journal.pone.0094614)
Supplement: Table S2 — The Quality of Observational Studies Assessed by Newcastle–Ottawa Scale. (DOCX) [file pone.0094614.s007.docx]

**Table S2: The Quality of Observational Studies Assessed by Newcastle – Ottawa Scale**

| **Study (Year)** | **Selection** | | | **Comparability** | | | **Outcome** | | | **Total score** |
| --- | --- | --- | --- | --- | --- | --- | --- | --- | --- | --- |
|  | **1** | **2** | **3** | **4** | **5A** | **5B** | **6** | **7** | **8** |  |
| **Gasparini M (2003) ^9^** | B (*) | A (*) | A (*) | B (0) | A (*) | B (*) | C (0) | A (*) | A (*) | 6 |
| **Molhoek SG (2004) ^29^** | B (*) | A (*) | A (*) | A (*) | No | B (*) | C (0) | A (*) | A (*) | 6 |
| **Leclercq C (2004) ^30^** | B (*) | A (*) | A (*) | A (*) | No | B (*) | B (*) | A (*) | B (*) | 7 |
| **Waggoner, A.D (2006) ^10^** | B (*) | A (*) | A (*) | A (*) | No | B (*) | A (*) | A (*) | A (*) | 7 |
| **Soliman, O. I (2007) ^31^** | B (*) | A (*) | A (*) | A (*) | A (*) | B (*) | C (0) | A (*) | A (*) | 8 |
| **D'Andrea, A (2007) ^32^** | A (*) | A (*) | C (0) | A (*) | No | B (*) | C (0) | A (*) | A (*) | 6 |
| **Vidal, B (2007) ^33^** | B (*) | A (*) | A (*) | A (*) | No | B (*) | B (*) | A (*) | A (*) | 7 |
| **Di Biase L (2008) ^11^** | A (*) | A (*) | A (*) | A (*) | A (*) | B (*) | B (*) | A (*) | A (*) | 8 |
| **Marsan, N. A (2009) ^34^** | A (*) | A (*) | C (0) | A (*) | No | A (*) | C (0) | A (*) | B (*) | 6 |
| **Boriani,G (2009) ^35^** | A (*) | A (*) | A (*) | A (*) | A (*) | B (*) | B (*) | A (*) | A (*) | 8 |
| **Zhang, Q (2009) ^36^** | B (*) | A (*) | A (*) | A (*) | A (*) | B (*) | C (0) | A (*) | B (*) | 7 |
| **Kazemi S.A (2009) ^37^** | B (*) | A (*) | C (0) | A (*) | No | B (*) | C (0) | A (*) | A (*) | 6 |
| **Mcleod CJ (2011) ^38^** | A (*) | A (*) | A (*) | A (*) | No | A (*) | B (*) | A (*) | A (*) | 7 |
| **Zaca, V (2011)^39^** | A (*) | A (*) | No | Yes | No | B (*) | C (0) | A (*) | B (*) | 6 |
