# Supplementary material for: Impact of Etiology on the Outcomes in Heart Failure Patients Treated with Cardiac Resynchronization Therapy: A Meta-Analysis
Source: PLoS One. 2014 Apr 14;9(4):e94614. doi: 10.1371/journal.pone.0094614 (PMC3986107; doi:10.1371/journal.pone.0094614)
Supplement: Table S3 — Characteristics of patients enrolled in Observational Studies. (DOCX) [file pone.0094614.s008.docx]

**Table S3: Characteristics of patients enrolled in Observational Studies**

| **Study**  **(Year)** | **Definition of ICM** | **Mean age** | | **Males (%)** | | **NYHA class*** | | **Mean QRS (ms)** | | **LVEF (%)** | |
| --- | --- | --- | --- | --- | --- | --- | --- | --- | --- | --- | --- |
|  |  | **ICM** | **NICM** | **ICM** | **NICM** | **ICM** | **NICM** | **ICM** | **NICM** | **ICM** | **NICM** |
| **Gasparini M(2003) ^9^** | NA | 67 | 65 | 92 | 63 | III/IV(83) | III/IV(80) | 169(31) | 177(28) | 30(7) | 30(7) |
| **Molhoek SG(2004) ^29^** | significant CAD( >50% stenosis) by CA | 65(10) | 64(11) | 88 | 68 | III (90) | III(80) | 175(29) | 178(29) | 21 (9) | 23(13) |
| **Leclercq C(2004) ^30^** | i) signiﬁcant CAD confirmed by CA, ii) prior MI  iii) prior CABG or PCI | 70(8) | 65(12) | 92 | 67 | III (69) | III (67) | 180(29) | 176(27) | 22(6) | 22(8) |
| **Waggoner, A.D(2006) ^10^** | Significant CAD (>50% stenosis) by CA | 63(11) | 60(12) | 95 | 66 | III or IV | III or IV | 180(20) | 180(30) | ≤ 35% | ≤35% |
| **Soliman, O. I(2007) ^31^** | NA | 59(11) | 59(10) | 77 | 63 | III (94) | III (92) | 169(29) | 172(27) | 19 (4) | 17(4) |
| **D'Andrea, A(2007) ^32^** | NA | 57(11) | 55(8) | 55 | 51 | III (82) | III (82) | 149(22) | 149(22) | 31(4) | 30(4) |
| **Vidal, B(2007) ^33^** | significant CAD(>50%stenosis) by CA | 69(7) | 69(8) | NA | NA | II-IV | II-IV | 140(28) | 154(29) | 28 (8) | 25(6) |
| **Di Biase L(2008)^11^** | significant CAD by CA | 71(9) | 63(13) | 86 | 61 | III/IV(86) | III/IV(88) | 207(94) | 162(90)† | 22 (8) | 22(8) |
| **Marsan, N. A(2009) ^34^** | NA | 65(10) | 67(17) | 90 | 80 | III (95) | III (97) | 143(28) | 153(33) | 25 (8) | 25(8) |
| **Boriani,G(2009) ^35^** | i) significant CAD(>75% stenosis) by CA, ii) prior MI, iii) prior PCI or CABG | 69(8) | 66(10) | 90 | 72 | III (67) | III(66) | 163(32) | 165(30) | 26 (7) | 26(7) |
| **Zhang, Q(2009) ^36^** | i) positive findings on CA ii) prior MI iii) underwent PCI | 65(12) | 64(13) | 75 | 73 | III (87) | III (85) | 131(31) | 137(37) | 27 (7) | 26(9) |
| **Kazemi S.A(2009) ^37^** | significant known CAD by CA | 59(10) | 56(11) | 85 | 60 | III or IV | III or IV | 137(22) | 137(22) | 21 (6) | 19(5) |
| **Mcleod CJ(2011) ^38^** | NA | 71(9) | 15(13) | 88 | 65 | III (50) | III(50) | 165(34) | 170(34) | 23(7) | 23(8) |
| **Zaca, V(2011) ^39^** | i) positive findings at CA ii) prior MI, iii) prior PCI or CABG | 66(8) | 67(8) | 74 | 67 | III (93) | III(93) | 176(27) | 180(28) | 25(6) | 24(6) |

* The values are given as the NYHA class (proportion of patients); †the value is given as numbers and proportion of patients (%); other values are given as mean (standardized deviation). CAD, coronary artery diseases; ICM, ischemic cardiomyopathy; LVEF, left ventricular ejection fraction; NA, not available; NICM, non-ischemic cardiomyopathy; NYHA, New York Heart Association.
